# CancerSupportSource®: validation of a revised multi-dimensional distress screening program for cancer patients and survivors

**DOI:** 10.1007/s00520-019-04753-w

**Published:** 2019-04-12

**Authors:** Joanne S. Buzaglo, Alexandra K. Zaleta, Shauna McManus, Mitch Golant, Melissa F. Miller

**Affiliations:** 1 Cancer Support Community, Research and Training Institute, 520 Walnut Street, Suite 1170, Philadelphia, PA 19106 USA; 2 Present Address: Concerto HealthAI, 501 Boylston Street 10th Floor, Boston, MA 02116 USA

**Keywords:** Cancer, Distress, Screening, Survivorship, Depression, Anxiety

## Abstract

**Purpose:**

To facilitate access to and provision of psychosocial care to cancer patients in the community, the Cancer Support Community (CSC) developed CancerSupportSource® (CSS), an evidence-based psychosocial distress screening program*.* The current study examined the psychometric properties and multi-dimensionality of a revised 25-item version of CSS, and evaluated the scale’s ability to identify individuals at risk for clinically significant levels of depression and anxiety.

**Methods:**

CSS development and validation were completed in multiple phases. Exploratory factor analysis was completed with 1436 individuals diagnosed with cancer to examine scale dimensionality, and nonparametric receiver operating characteristic (ROC) curve analyses were used to determine scoring thresholds for depression and anxiety risk scales. Internal consistency reliability and convergent and discriminant validity were also examined. Confirmatory factor analysis and intraclass correlation coefficients were subsequently calculated with a separate sample of 1167 individuals to verify the scale factor structure and examine test–retest reliability.

**Results:**

Five factors were identified and confirmed: (1) emotional well-being, (2) symptom burden and impact, (3) body image and healthy lifestyle, (4) health care team communication, and (5) relationships and intimacy. Psychometric evaluation of the total scale and factors revealed strong internal consistency reliability, test–retest reliability, and convergent and divergent validity. Sensitivity of CSS 2-item depression and 2-item anxiety risk scales were .91 and .92, respectively.

**Conclusions:**

Results indicate that CancerSupportSource is a reliable, valid, multi-dimensional distress screening program with the capacity to screen for those at risk for clinically significant levels of depression and anxiety.

## Background

It is estimated that there will be more than 1,700,000 new cases of cancer in the U.S. in 2018 (1). Many survivors will receive state-of-the-science biomedical treatment, but will not receive care that adequately assesses or addresses their psychosocial concerns (2). This is problematic in part because distress and unmet psychosocial needs can have adverse implications for health outcomes, health service use, treatment adherence, and health care costs (3, 4). Further, there is research to suggest that treating and addressing cancer patients’ distress can lead to improved overall health and reduced health care costs (5, 6).

Given the immediate and long-range implications of distress among persons with cancer, the Institute of Medicine (7) and the National Comprehensive Cancer Network (8) have recognized that screening, referral, and follow-up for psychosocial concerns are critical to ensuring quality cancer care for the whole patient. Further, the Commission on Cancer of the American College of Surgeons released accreditation standards requiring practices to screen cancer patients for psychosocial distress (9), and the American Society of Clinical Oncology recommends regular screening of adults with cancer for depression and anxiety (10). Providing patients and clinicians with a distress screening and referral program that is comprehensive, easily accessible (i.e., by Internet), and patient-centered in terms of resource/referral preferences can offer benefit to patients, and potentially, the broader system of care. A number of validated measures exist to assess distress (11–13) and identify areas of unmet need (14, 15). Among those tools that assess patient preference for help, few ask the patient to delineate the type of help they wish to receive or have the ability to integrate across electronic systems via a web-based platform. Even fewer were developed and validated for use in community-based care settings, despite the fact that a majority of cancer patients are treated in community cancer centers, hospitals, and private practices (16).

The Cancer Support Community (CSC) developed CancerSupportSource® (CSS), a web-based psychosocial distress screening, follow-up, and referral program to assist community-based cancer centers in meeting distress screening accreditation standards linked to patient-centered cancer care (17), and to facilitate access to and provision of psychosocial care to cancer patients in the community. The initial theoretical framework and item content of CSS are grounded in the critical domains of psychosocial, practical, and physical needs (7, 18). Further, the design of the screening program reflects CSC’s model of patient empowerment (19). In this model, patients are supported and encouraged to identify their needs and play an active role in partnering with their health care providers, make important lifestyle changes, and enhance their ability to access information and resources that can address their needs.

Preliminary development of CSS has been previously described (17). Scale content was informed by patient focus groups and cognitive interviews, as well as expert input; initial psychometric validation was conducted among CSC’s community-based affiliate sites with a convenience sample of 251 cancer patients. In completing CSS, respondents are asked to rate their level of concern for 25 items, and in its full implementation, respondents also indicate desired help (additional information or talk to a staff member) and are provided tailored, automated referrals to support resources and follow-up care. The preliminary scale demonstrated high internal consistency reliability (Cronbach’s *α* = .92) and moderate to strong concurrent validity via associations with the FACT-G (*r* = − .70, *p* < .001), National Comprehensive Cancer Network (NCCN) distress thermometer (*r* = .62, *p* < .001), and CES-D (*r* = .69, *p* < .001). Items most highly correlated with the CES-D included feeling sad or depressed (*r* = .72, *p* < .001) and feeling lonely or isolated (*r* = .72, *p* < .001); these associations informed early conceptualization of a depression risk screening subscale. In the initial validation, items most frequently endorsed for additional follow-up with staff included worrying about the future and what lies ahead, feeling sad or depressed, and eating and nutrition. Intraclass correlation coefficients (ICCs) were used to examine test–retest reliability with a time interval between administrations of approximately 2 hours; items with ICCs < .75 (*n* = 9) were revised to assist with clarity and interpretability, resulting in a revised 25-item version of CSS.

The aims of the current study were to (1) examine the psychometric properties and multi-dimensionality of a revised 25-item version of CSS and (2) examine the scale’s ability to identify individuals at risk for clinically significant levels of depression and anxiety.

## Methods and results

From March 2013 to December 2017, validation of CSS was completed in two phases: (1) exploratory factor analysis and measure validation and (2) confirmatory factor analysis and test–retest reliability.

### Phase I—Exploratory Factor Analysis and measure validation

#### Participants

Data were collected through the Cancer Support Community’s Cancer Experience Registry® (CER), an online, community-based research initiative examining the social and emotional impact of cancer. Recruitment for the registry occurred through CSC’s network of community-based affiliates/chapters, online communities, CSC’s toll-free Cancer Support Helpline®, other cancer advocacy organizations, and social media. Ethical and Independent Review Services (E&I, Independence, MO) served as the IRB of record. All procedures performed in studies involving human participants were in accordance with the ethical standards of the institutional and/or national research committee and with the 1964 Helsinki declaration and its later amendments or comparable ethical standards. Informed consent was obtained from all individual participants included in the registry. Eligibility to participate in the CER includes being 18 years of age or older and having ever been diagnosed with cancer. Potential participants for phase I of the current study participated in the CER survey from March 2013 to December 2016 (*n =* 4668).

#### Procedures

Participants provided demographic information (age, gender, race, ethnicity, education, employment status, and household income). Self-reported cancer diagnosis, stage at diagnosis, and time since diagnosis and treatments received were also obtained. We excluded survey participants living outside the U.S. (*n* = 288) and those missing > 50% of CSS distress items (< 1% excluded); those included also completed at least one comparative measure scale, resulting in a total analytic sample size of 1436 (Table 1). Compared to the sample of registrants who provided basic background information but did not complete the full length of the survey (*n =* 2944), the analytic sample for this study (*n =* 1436) was more likely to be male (28% vs. 17%, *p <* .001) and older (mean age = 58 years vs. 55 years, *p <* .001).

**Table 1:** Descriptive characteristics of the sample

		Phase I data (*N* = 1436)		Phase II data (*N* = 1167)	
		*M*/*n*	SD/%	*M*/*n*	SD/%
Age		58.4 (*n* = 1309)	11.1	58.4 (*n* = 1104)	12.2
Sex	Female	1035	72%	791	69%
	Male	401	28%	357	31%
Race	White	1291	90%	964	83%
	African American	64	4%	89	8%
	Asian	18	1%	13	1%
	American Indian or Alaskan Native	5	< 1%	4	< 1%
	Native Hawaiian or Pacific Islander	4	< 1%	1	< 1%
	Multiple races	25	2%	22	2%
Hispanic or Latino/a		40	3%	68	6%
Education	No college	196	14%	210	18%
	Some college	293	20%	279	24%
	College degree	534	37%	391	34%
	Graduate or professional degree	402	28%	255	22%
Annual income	< $20 K	108	8%	156	34%
	$20–39 K	207	14%	168	14%
	$40–59 K	181	13%	143	12%
	$60–79 K	145	10%	119	10%
	$80–99 K	136	9%	95	8%
	$100 K+	300	21%	199	17%
	Prefer not to share	283	20%	242	21%
Employment	Full-time	450	31%	342	29%
	Part-time	138	10%	97	8%
	Retired	447	31%	350	30%
	Disability	256	18%	243	21%
	Unemployed	110	8%	91	8%
Cancer diagnosis (most recent)	Breast	504	35%	365	31%
	Hematologic	493	34%	129	11%
	Lung	65	5%	103	9%
	Prostate	54	4%	173	15%
	Ovarian	50	3%	45	4%
	Colorectal	44	3%	60	5%
	Melanoma	34	2%	25	2%
	Head and neck	20	1%	36	3%
	Endometrial	18	1%	25	2%
	Sarcoma	11	1%	17	2%
	Stomach	8	1%	17	2%
	Other	277	19%	347	30%
Years since diagnosis		4.6 (*n* = 1430)	5.3	4.4 (*n* = 1059)	6.1
Stage	0	93	6%	60	5%
	I	295	21%	201	19%
	II	319	22%	220	19%
	III	319	22%	207	18%
	IV	194	14%	167	14%
	Other	45	3%	35	3%
	Do not know	154	11%	159	14%
Treatment history	Current chemotherapy	301	21%	278	24%
	Current radiation therapy	62	4%	202	17%
	Current hormonal therapy	217	15%	213	18%
	Ever chemotherapy	1029	72%	696	60%
	Ever radiation therapy	629	44%	522	45%
	Ever hormonal therapy	309	22%	285	24%
	Past surgery	878	61%	771	66%

Other cancer diagnoses included cervical, pancreatic, bladder, esophageal, kidney/renal cell, brain, testicular, and vaginal, among others

#### Measures

##### CancerSupportSource

Cancer-related distress was assessed using the revised 25-item version of CancerSupportSource (CSS-25). Patients rated their level of concern (0 = not at all, 1 = slightly, 2 = moderately, 3 = seriously, 4 = very seriously) for each item plus one additional exploratory item assessing concerns about thinking clearly; request for follow-up services was not assessed for the current study. Completion of CSS takes approximately 5 to 10 min. A total distress score was calculated as the sum of item ratings. For those participants missing CSS responses, the mean of available items for each individual was used to impute an overall score. The square root of the total distress score was approximately normally distributed. A 2-item depression risk score was calculated by summing two items (feeling sad or depressed; feeling lonely or isolated); a 2-item anxiety risk score was calculated by summing two items (feeling nervous or afraid; worrying about the future and what lies ahead).

##### PROMIS-29

Participant self-reported symptoms and functioning were examined using the Patient-Reported Outcomes Measurement Information System—29 (PROMIS-29 v2.0), a collection of 4-item short forms plus one item assessing pain intensity (20). Five domains assess symptoms with higher scores corresponding to worse symptomatology (depression, anxiety, pain interference, fatigue, sleep disturbance) and two assess function with lower scores corresponding to worse functioning (physical function, ability to participate in social roles and activities). Participants rate each item with reference to the past 7 days; function scales have no timeframe specified. Scale scores are converted to standardized *T* scores (mean = 50, SD = 10); normative reference groups are the U.S. general population, except sleep disturbance, where the reference is based on the U.S. general population and a clinical sample which was generally more enriched for chronic illness. In this case, a score of 50 likely represents somewhat sicker people than the general population.

##### General health

Participants rated their perceived level of general health (1 = poor; 5 = excellent) (21).

##### Analysis

Data analysis was conducted using Stata 14.2 (22) and R 3.4.0 (23), with GPArotation (24) and psych (25) R packages. Descriptive statistics were calculated for socio-demographic, disease, distress, general health, and patient-reported outcome variables. One sample *t* tests of means were used to compare means for PROMIS scales to the national average of *T* = 50 for adults in the U.S. Exploratory factor analysis with direct oblique rotation and principal axis factoring (PAF) extraction was used to analyze patterns in the inter-item correlation matrix and to assess the dimensionality (number of factors) needed to represent the variability in the data. Both parallel analysis and Cattell’s scree test, as well as examination of fit indices, were performed in order to determine the number of optimal factors for the scale (26). We used factor loadings to indicate the relevance of the variables to each factor. Items were considered to cross-load if the item had a factor loading > 0.4 on more than one factor, and the difference between factor loadings was < 0.2. Items that cross-loaded were not included in the final factor structure. Internal consistency reliability was estimated using Cronbach’s alpha coefficient (27, 28). Nonparametric receiver operating characteristic (ROC) curve analyses were used to determine scoring thresholds for depression and anxiety risk scales, using PROMIS depression (*T* ≥ 60) and anxiety (*T* ≥ 62) scales as criterion scores (29, 30) as these correspond to conventional cutoffs for clinical risk significance using the PHQ-9 and GAD-7 legacy instruments (31–33). We assessed convergent validity with Pearson correlation coefficients (34). Discriminant validity was determined using the known-group validation method; two sample *t* tests were used to verify whether summary scores could differentiate according to demographic and clinical variables: gender, currently receiving treatment for cancer, and time since diagnosis. Cohen’s *d* was used to estimate effect size between groups using the square root of the distress score.

## Results

### Clinical description of the sample

#### Participant socio-demographics

Participants were predominantly female (72%), White (90%), an average of 58 years of age (range = 19–87), and 4.6 years from their first cancer diagnosis (range = < 1 to 52 years; Table 1). The sample comprised a wide range of cancer patients and survivors representing various cancer diagnoses.

#### Cancer-related distress

The items of greatest distress (rated moderately to very seriously concerned) were the following: eating and nutrition (57.8%), exercising (50.0%), worrying about the future (47.6%), feeling too tired (46.9%), and health insurance or money worries (42.1%; Table 2). Of note, 20.3% of participants reported serious to very serious concern about thinking clearly; this new CSS item was selected to replace an infrequently endorsed item (9.1% indicated serious or very serious concern) assessing interest in complementary and alternative treatments, and was also used in calculating CSS total distress score for the 25-item scale.

**Table 2:** Factor loadings and communalities based on exploratory factor analysis

Item	Mean/SD	% ≥ 3	% ≥ 2	Factor 1	Factor 2	Factor 3	Factor 4	Factor 5	Communalities
Factor 1: Emotional well-being									
Feeling nervous or afraid	0.93/1.06	10.4	24.7	*.82*	− .05	.08	− .06	.11	.71
Feeling sad or depressed	1.08/1.15	12.7	30.6	*.77*	.10	− .02	.06	− .06	.71
Worrying about the future and what lies ahead	1.63/1.26	24.7	47.6	*.77*	.02	.08	− .03	.04	.67
Feeling lonely or isolated	0.95/1.20	13.5	26.9	*.71*	.06	− .03	.17	− .07	.68
Finding meaning and purpose in life	0.97/1.21	13.3	28.3	*.56*	.00	.06	.23	.03	.58
Worrying about family, children, and/or friends	1.21/1.19	15.7	36.1	*.43*	.13	.09	.06	.15	.46
Health insurance or money worries	1.46/1.41	25.5	42.1	*.39*	.18	.04	.07	.08	.37
Feeling irritable	1.10/1.09	11.7	33.6	*.34*	.06	.17	.20	.11	.46
Factor 2: Symptom burden and impact									
Pain and/or physical discomfort	1.21/1.18	15.5	35.6	.04	*.77*	− .03	− .01	.04	.62
Moving around (walking, climbing stairs, lifting, etc.)	1.28/1.22	16.9	39.8	− .16	*.74*	.14	.01	.08	.59
Feeling too tired to do the things that you need or want to do	1.55/1.28	25.2	46.9	.16	*.69*	.09	.06	− .10	.72
Managing side effects of treatment (nausea, swelling, etc.)	0.97/1.18	13.5	28.2	.15	*.55*	.00	− .01	.15	.50
Changes or disruptions in work, school, or home life	1.21/1.27	17.2	37.0	.25	*.45*	.01	.15	.04	.56
Thinking clearly (e.g., “chemo brain,” “brain fog”)	1.32/1.29	20.3	40.2	.15	*.39*	.10	.06	.07	.38
Sleep problems	1.37/1.18	18.7	41.0	.25	*.30*	.07	.10	.02	.37
Transportation to treatment and appointments	0.36/0.82	3.6	9.7	.16	*.30*	− .07	.07	.27	.33
Factor 3: Body image and healthy lifestyle									
Exercising and being physically active	1.59/1.26	26.0	50.0	− .06	.28	*.61*	.09	− .02	.65
Recent weight change (gain or loss)	1.03/1.23	13.8	30.1	.15	.04	*.60*	− .01	.03	.50
Body image and feelings about how you look	1.26/1.24	17.0	38.0	.27	.01	*.53*	.06	− .04	.53
Eating and nutrition	1.74/1.31	30.2	57.8	.01	− .11	*.49*	.31	.06	.37
Factor 4: Health care team communication									
Communicating with your doctor	0.73/1.16	11.2	21.3	.01	.19	.04	*.48*	.15	.45
Making a treatment decision	0.91/1.17	12.1	26.9	.36	.15	− .02	*.43*	.00	.53
Factor 5: Relationships and intimacy									
Problems in your relationship with your spouse/partner	0.71/1.12	10.0	19.5	.00	− .03	− .01	.03	*.82*	.64
Intimacy, sexual function and/or fertility	1.09/1.27	16.5	33.5	.04	.05	.12	− .03	*.54*	.43

Italics denote items corresponding to each factor. “Tobacco or substance use - by you or someone in your household,” did not have a factor loading > .30 on any of the five factors but was retained in the final 25-item scale due to its significance for clinical risk assessment

#### Patient-reported outcomes

Among the PROMIS-29 scales (mean ± SD, *p*-value; % scoring > 1SD than reference group) (35), participants reported poorer quality of life than the general U.S. population with respect to fatigue (53.6 ± 11.4, *p* < .001, *n* = 1398; 30.3%), anxiety (53.4 ± 10.5, *p* < .001, *n* = 1407; 28.7%), pain interference (52.2 ± 10.0, *p* < .001, *n* = 1158, 24.3%), physical function (45.7 ± 9.0, *p* < .001, *n* = 1417; 29.6%), and ability to participate in social roles (48.8 ± 10.2, *p* < .001, *n* = 1160; 18.8%). Participants also reported worse sleep disturbance (52.0 ± 8.3, *p* < .001, *n* = 1375; 13.2%) than a reference group that included both the general U.S. population and a clinical sample. Average depression scores were not significantly different from the general U.S. population (50.4 ± 9.6, *p* = .12, *n* = 1413, 19.2%).

#### General health

Mean (SD) rating for general health was 3.18 (1.00; *n* = 1138); 9.0% indicated their general health was excellent and 24.3% indicated fair or poor.

### Psychometric analysis of CSS-25

#### Exploratory factor analysis

Examination of scree plot and parallel analysis, as well as fit indices for a range of models (three to seven factors), supported a five-factor solution that explained 52% of the total variance in the 25 distress items and produced the strongest model statistics (SRMR = 0.02; RMSEA = 0.06; see Table 2). The first factor explained 18% of the variance in distress and included eight items assessing emotional well-being. The second factor explained 13% of the variance and included eight items assessing symptom burden and impact. The remaining factors included items assessing body image and healthy lifestyle, health care team communication, and relationships and intimacy. Tobacco or substance use did not have a factor loading > 0.30 on any of the five factors.

#### Internal consistency reliability

Internal consistency reliability of the total scale score and factors were evaluated using Cronbach’s alpha (36) (Table 3). The factors demonstrated moderate to large inter-correlations, but were not redundant. Cronbach’s alpha for the full 25-item scale was .94.

**Table 3:** Scale and factor descriptive characteristics, inter-correlations, and internal consistency reliability

	No. of items	Mean/SD	Inter-correlations					Internal consistency reliability (α)
			F1	F2	F3	F4	F5	
Total distress score (CSS-25)	25	27.9/19.2	.92	.90	.79	.70	.64	.94
F1. Emotional well-being	8	1.17/0.93	–	.73	.63	.60	.57	.90
F2. Symptom burden and impact	8	1.16/0.86		–	.64	.61	.49	.87
F3. Body image and healthy lifestyle	4	1.41/0.97			–	.47	.45	.77
F4. Health care team communication	2	0.82/1.00				–	.37	.63
F5. Relationships and intimacy	2	0.90/1.05					–	.68

Cronbach’s alpha reported for internal consistency reliability. Full-scale Cronbach’s *α* = .94

#### Receiver operating characteristic curve analysis

Using a PROMIS depression score of ≥ 60 to indicate risk for clinical levels of depression, a score of ≥ 3 on the 2-item CSS depression risk scale yielded a sensitivity and specificity of 91.4% and 79.5%, respectively (AUC = .923; Table 4). Using a PROMIS anxiety score of ≥ 62 to indicate risk for clinical levels of anxiety, a score of ≥ 3 on the 2-item CSS anxiety scale yielded a sensitivity and specificity of 91.8% and 70.9%, respectively (AUC = .903). Based on a cutoff score of 3 for each CSS risk scale, 33.9% of participants were at risk for clinically significant levels of depression and 42.9% at risk for clinically significant levels of anxiety.

**Table 4:** Percentages for sensitivity and specificity for CancerSupportSource depression and anxiety risk scales

CSS risk score	PROMIS–29 comparison measure	AUC	Cutoff	Sensitivity	Specificity
Depression risk scale	Depression scale, *T* ≥ 60	.923	2	97.0	63.2
			3	91.4	79.5
			4	79.4	89.3
Anxiety risk scale	Anxiety scale, *T* ≥ 62	.903	2	98.0	48.2
			3	91.8	70.9
			4	81.3	85.2

*AUC* area under the curve

#### Convergent validity

The mean (SD) total CSS distress score for 25 items was 27.9 (19.1; median = 25; range 0–99). Total distress was moderately to strongly correlated with PROMIS-29 scales (*r*s -.67 to .70, *p*s < .001). The CSS depression risk scale was strongly correlated with PROMIS depression (*r* = .79, *p* < .001), and the CSS anxiety risk scale with PROMIS anxiety (*r* = .74, *p* < .001; Table 5). All five CSS factors were associated with PROMIS scales, with stronger associations exhibited between similar domains. The first factor encompassing emotional well-being was most highly correlated with PROMIS depression and anxiety scales (*r*s = .77 and .76, *p*s < .001). The second factor encompassing concerns about symptom burden and impact was most highly correlated with PROMIS fatigue, pain interference, and physical and social function scales (*r*s − .74 to .72, *p*s < .001). The third factor, body image and healthy lifestyle, was most highly correlated with PROMIS fatigue and social function scales (*rs* = .49 and − .49, *ps* < .001). The fourth factor, health care team communication, was most strongly associated with the PROMIS anxiety scale (*r* = .45, *p* < .001), and the fifth, relationships and intimacy, with the PROMIS depression scale (*r* = .42, *p* < .001).

**Table 5:** Pearson correlations between CSS, PROMIS validation measures, and general health ratings

	Cronbach’s alpha	Mean/SD	PROMIS scales							
			Depression	Anxiety	Social function	Physical function	Fatigue	Sleep disturbance	Pain interference	General health
			Pearson correlations							
Total distress score (CSS-25)	.94	27.9/19.1	.70	.69	− .67	− .52	.67	.49	.58	− .46
Depression risk score	.83	2.02/2.16	.79	.70	− .52	− .35	.53	.41	.41	− .38
Anxiety risk score	.83	2.55/2.15	.69	.74	− .48	− .32	.49	.37	.38	− .38
F1. Emotional well-being	.90	1.17/0.93	.77	.76	− .56	− .38	.57	.45	.46	− .41
F2. Symptom burden and impact	.87	1.16/0.86	.58	.58	− .74	− .64	.72	.52	.69	− .50
F3. Body image and healthy lifestyle	.77	1.41/0.97	.44	.44	− .49	− .38	.49	.35	.40	− .27
F4. Health care team communication	.63	0.82/1.00	.42	.45	− .40	− .31	.38	.23	.36	− .28
F5. Relationships and intimacy	.68	0.90/1.05	.42	.41	− .39	− .25	.34	.27	.29	− .27

All *p*s < .001 for Pearson correlations

#### Discriminant validity

Several group comparisons supported known-group validity. The CSS total distress score was significantly (*t* = 7.37, *p* < .001) higher among those in active cancer treatment (*n* = 770) than among those who were not (*n* = 653; Cohen’s *d* = 0.39) and among those who were within 5 years of their cancer diagnosis (*n* = 922) than among those who were beyond 5 years (*n* = 508; *t* = 5.79, *p* < .001; Cohen’s *d* = 0.32), consistent with a small to medium value of *d*. Female participants reported more distress than male participants (Cohen’s *d* = 0.21; *t* = − 3.60, *p* < .001). The square root of the total distress score was inversely associated with age (*r* = − .30, *p* < .001, *n* = 1309) and time since diagnosis (*r* = − .13, *p* < .001, *n* = 1430).

### Phase II—Confirmatory Factor Analysis and test–retest reliability

#### Participants

Participant eligibility was the same as phase I of the study (see above). From January to December 2017, a separate sample of 1457 cancer patients and survivors participated in the survey.

#### Procedures and measures

Procedures and measures were identical to phase I of the study. We excluded survey participants living outside the US (*n* = 28) and limited the sample to those who answered at least 50% of the distress items and completed at least one quality of life scale, resulting in a total analytic sample size of 1167 (Table 1). Compared to the sample of registrants who provided basic background information but did not complete the full length of the survey (*n =* 290), the analytic sample for this phase of the study (*n =* 1167) was more likely to be White (84% vs. 65%, *p <* .01) and, on average, older (58.4 vs. 55.7 years *p <* .01). Additionally, a subset of participants (*n* = 249) was asked to complete the revised 25-item CancerSupportSource a second time at the end of the survey to examine test–retest reliability with a time interval between test administrations of approximately 30 to 90 min. CSS was administered before and after multiple questionnaires in order to reduce the effect of memory. The socio-demographic and clinical history variables for this subset of participants did not differ significantly from other phase II participants.

#### Analyses

Confirmatory factor analysis was conducted with maximum likelihood factor extraction and listwise deletion of missing data. The factor loadings for the first indicator in each factor were fixed to 1.0, and we allowed for correlations between factors, using a target matrix for our rotation method with the highest loading factors from the exploratory factor analysis (EFA) specified (37). Both absolute fit indices and relative fit indices were used to measure goodness of fit (38). Test–retest reliability was measured with intraclass correlation coefficients.

#### Results

The five-factor model explained 53% of the variance and again demonstrated good fit (RMSEA = .067; SRMR = .042; CFI = .922; *χ*^2^(242) = 1132.59, *p* < .001). All items demonstrated stability, with equivalent or higher loadings on their respective factors. Full scale test–retest reliability was .91, while all individual scales ICCs were ≥ .78, exceeding the threshold of .75 for excellent (39) test–retest reliability (emotional well-being = .91; symptom burden and impact = .91; relationships and intimacy = .85; health care team communication = .79; body image and healthy lifestyle = .78).

## Discussion

CancerSupportSource (CSS) is a reliable, valid, multi-dimensional distress screening program with the capacity to identify those at risk for significant anxiety and depression, known indicators of poor health outcomes (40–43). The current study findings provide the psychometric foundation to support the use of CSS in community-based and hospital-based oncology settings, and CSS is currently implemented across a network of community-based cancer support facilities (Cancer Support Community, Gilda’s Club) as well as oncology practices and hospital cancer centers nationwide. The tool demonstrates strong internal consistency reliability and test–retest reliability, a factor structure that is replicable, and adequate convergent and divergent validity. Additionally, the depression and anxiety risk scales demonstrate high sensitivity coupled with adequate specificity. When implemented in oncology practice, this tool fulfills the American College of Surgeons Commission on Cancer patient-centered standards for distress screening (9), NCCN Guidelines for distress management (8), and the American Society of Clinical Oncology (ASCO) Quality Oncology Practice Initiative (QOPI) certification standards (44).The strengths of this study include that it is a community-based research program, currently validated across a broad sample of survivors representing diverse cancer care settings and geographic regions. Further, our results support that CSS is a relatively brief but multi-dimensional tool, which can assist providers in developing a personalized, integrated care plan. Additionally, our results provide psychometric support for the inclusion of depression and anxiety risk scales. These scales provide a brief screening of individuals at risk for clinically significant levels of depression and anxiety, without additional administration burden to patients or providers, an important consideration given that distress screening uptake continues to be a challenge across cancer care (45). Integrated distress and risk screening can be beneficial in community-based settings in particular by facilitating a first step in triaging patients to appropriate levels of care, as determined by each care system in which it is implemented.

Limitations of the current study include self-selected samples of participants who have Internet access, are predominantly female, White, fairly educated, and include a substantial proportion of individuals with breast and hematologic cancers. These limitations impact the study results’ generalizability to a more diverse socio-economic population. This sample is not representative of all cancer patients and survivors across the U.S.; however, it is a representative of those who seek social and emotional support in the community. Future work is required to understand the effectiveness of implementing the tool and to evaluate its applicability and cultural sensitivity among other diverse samples and settings. Additionally, the time interval for test–retest reliability was relatively short, although CSS was administered after multiple questionnaires in order to reduce the effect of memory. Internal consistency reliability for two factors was .60 < *α* < .70, which may in part reflect the limited number of items that loaded on these constructs; however, these factors were retained due to their relevance to needs assessment for cancer patients and survivors. The depression and anxiety risk cutoff scores were developed in comparison to the PROMIS-29; future evaluation and testing of these risk scales will consider integration of structured clinical interviews. The current study did not examine participants’ desired follow-up on items (e.g., information or talking to a staff member); these will be explored in future implementation studies.

Distress screening is the necessary first step in a comprehensive assessment of the patient experience, functioning, and unmet needs related to emotional well-being, symptom burden and impact, body image and healthy lifestyle, health care team communication, and relationships and intimacy. This study focuses on the validation and psychometric evaluation of CancerSupportSource and its content. Future work will focus on implementation of distress screening, uptake of referrals and follow-up, and the impact of distress screening on quality- and cost-related outcomes in diverse settings. Particularly in settings where high socio-economic need exists and health and support services are remote, efforts are needed to evaluate innovative approaches that leverage technology and community resources to deliver tailored interventions that meet patient and family needs. Such efforts will support the overarching goal of examining a stepped care approach to delivering psychosocial care tailored to the individualized needs of cancer survivors over time.

### Acknowledgements

Support for the Cancer Experience Registry was provided by AbbVie, Inc.; Amgen Oncology; Astellas Pharma US, Inc.; AstraZeneca; Bayer HealthCare; Boehringer Ingelheim; Bristol-Myers Squibb; Celgene Corporation; Genentech, Inc.; Janssen Oncology; Jazz Pharmaceuticals; Lilly Oncology; Novartis; Pfizer Oncology; Pharmacyclics, Inc.; and Takeda Oncology.
